# Modelling the Influence of Major Baltic Inflows on Near-Bottom Conditions at the Entrance of the Gulf of Finland

**DOI:** 10.1371/journal.pone.0112881

**Published:** 2014-11-13

**Authors:** Gennadi Lessin, Urmas Raudsepp, Adolf Stips

**Affiliations:** 1 Plymouth Marine Laboratory, Prospect Place, The Hoe, Plymouth, United Kingdom; 2 Marine Systems Institute, Tallinn University of Technology, Tallinn, Estonia; 3 European Commission, Joint Research Centre, Institute for Environment and Sustainability, Water Research Unit, Ispra, Italy; University of Vigo, Spain

## Abstract

A coupled hydrodynamic-biogeochemical model was implemented in order to estimate the effects of Major Baltic Inflows on the near-bottom hydrophysical and biogeochemical conditions in the northern Baltic Proper and the western Gulf of Finland during the period 1991–2009. We compared results of a realistic reference run to the results of an experimental run where Major Baltic Inflows were suppressed. Further to the expected overall decrease in bottom salinity, this modelling experiment confirms that in the absence of strong saltwater inflows the deep areas of the Baltic Proper would become more anoxic, while in the shallower areas (western Gulf of Finland) near-bottom average conditions improve. Our experiment revealed that typical estuarine circulation results in the sporadic emergence of short-lasting events of near-bottom anoxia in the western Gulf of Finland due to transport of water masses from the Baltic Proper. Extrapolating our results beyond the modelled period, we speculate that the further deepening of the halocline in the Baltic Proper is likely to prevent inflows of anoxic water to the Gulf of Finland and in the longer term would lead to improvement in near-bottom conditions in the Baltic Proper. Our results reaffirm the importance of accurate representation of salinity dynamics in coupled Baltic Sea models serving as a basis for credible hindcast and future projection simulations of biogeochemical conditions.

## Introduction

The Baltic Sea is a brackish inland water body having a limited water exchange with the North Sea through narrow and shallow Danish Straits. It receives large freshwater runoff and riverine nutrient loads, which in 2006 comprised 638,000 t of total nitrogen and 28,370 t of total phosphorus [1]. After the collapse of the Soviet Union and consequent socio-economic changes in the region in the 1990s, a considerable reduction of nutrient discharge to the sea from agricultural runoff and industrial pollution took place. The total decrease was approximately 35% for both phosphorus and nitrogen [2]. However, eutrophication resulting from direct and indirect input of nutrients is still considered one of the major environmental problems in the sub-basins of the Baltic Sea [3], [4], with the Gulf of Finland being the most eutrophied of them [5], [6]. The Gulf of Finland, a relatively narrow (50–135 km) basin in the eastern part of the Baltic Sea, is about 400 km long. Its maximum depth decreases from 80–100 m at the entrance to 20–30 m in the eastern part which receives the Neva river discharge. The Gulf of Finland receives about 2 times larger nitrogen and 3 times larger phosphorus inputs than the Baltic Sea in relation to the surface area [7]. Despite the considerable reduction in nutrient discharge to the Baltic Sea as a whole, there have been repeated reportings of the occurrence of near-bottom anoxic conditions and elevated phosphate concentrations in the Gulf of Finland since the beginning of the 2000s [8]–[11].

The hydrophysical and biogeochemical status of the Baltic Sea and its sub-basins is to a large extent shaped by external forcing, comprising direct interaction with the atmosphere, freshwater runoff and nutrient discharge from the surrounding land as well as interactions between the sub-basins and interactions at the open boundary [12]. The latter is of a special importance because episodic barotropic inflows – termed Major Baltic Inflows (MBIs) – of highly saline and oxygenated water are an important mechanism of deep water renewal and displacement in the Baltic Sea [13]. Although inflows of different intensity take place more or less regularly, according to the classification based on the duration of inflow and mean vertical salinity as proposed by [14] only 7 MBIs that were observed during the period between 1880–2007 are classified as “very strong” [15]–[17] [13]. One of these very strong inflows occurred in 1993, terminating an unusually lengthy stagnant period that lasted for more than a decade. This breakdown of stagnant conditions took place at the beginning of the period of significant reduction of nutrient loads to the Baltic Sea, which raises the question as to what extent the occurred MBIs are responsible for the changes in biogeochemical conditions in the Gulf of Finland during the following decades.

A major part of the research on the influence of MBIs on hydrophysical and biogeochemical conditions in the Baltic Sea concentrated on the deep basins of the Baltic Proper, e.g. [18]–[20]. In the Gulf of Finland, research on the effect of MBIs was mainly focused on relating the observed physical and biogeochemical/ecological conditions in the Gulf to the timing and extent of inflows to the Baltic Proper. For instance, the influence of changes in salinity and density stratification on oxygen concentrations in the second half of the 20^th^ century was assessed based on long-term measurement data [21],[22]. A combination of satellite imagery and *in situ* data was used to propose that the MBI of 1993 was responsible for the expansion of *Nodularia spumigena* blooms to the central and eastern Gulf of Finland after 1994 [8]. This type of connection between a physical process and the ensuing ecosystem changes is of special importance, since harmful algae blooms remain one of the major environmental problems for the whole Baltic Sea and for the Gulf of Finland in particular [23],[24].

In the present study, a coupled three-dimensional hydrodynamic-biogeochemical model was applied to study the changes in salinity, nutrients and oxygen in the northern part of the Baltic Proper and the western Gulf of Finland in relation to the MBIs that had occurred since the beginning of the 1990s. The main goals of this modelling study were:

a) to realistically simulate the changes in near-bottom salinity, nutrients and oxygen dynamics in the northern Baltic Proper and the western Gulf of Finland during 1991–2009 by means of a 3D hydrodynamic-biogeochemical model;
b) to estimate the role of MBIs in shaping the hydrophysical and biogeochemical conditions in the northern Baltic Proper and the western Gulf of Finland by comparing the realistic results to an experimental run where MBIs were suppressed.

## Model Description and Setup

Model simulations were performed using the hydrodynamic model GETM (General Estuarine Transport Model, www.getm.eu, accessed 2014 Oct 24) coupled with the ERGOM (Ecological Regional Ocean Model, www.ergom.net, accessed 2014 Oct 24) biogeochemical model. GETM is a three-dimensional free-surface hydrodynamic model, which solves the primitive equations of water dynamics with a mode splitting technique on the Arakawa C-grid using Boussinesq and hydrostatic approximations [25],[26]. GOTM (General Ocean Turbulence Model, www.gotm.net, accessed 2014 Oct 24) is coupled to GETM to resolve vertical mixing using the k-ε turbulence closure scheme [27]. The ERGOM model version applied in this study contains 12 state variables: three phytoplankton groups (diatoms, flagellates and nitrogen-fixing cyanobacteria), nitrate, ammonium, phosphate, bulk zooplankton, detritus, dissolved oxygen, sediment detritus, iron-bound phosphorus in water and in the sediments. ERGOM uses nitrogen as a model currency. Nitrate, ammonium and phosphate are taken up by phytoplankton in accordance with Redfield nitrogen to phosphorus ratio 16∶1. It is assumed that cyanobacteria are able to fix atmospheric nitrogen and are limited only by availability of phosphate. Ammonium and phosphate are released by respiration, excretion and detritus mineralisation. In the presence of oxygen, part of the ammonium is converted to nitrate through the process of nitrification. Under anaerobic conditions, and in the presence of nitrate, detritus is oxidised by reducing nitrate to dinitrogen gas which leaves the system. Under anaerobic conditions and depleted nitrate, hydrogen sulphide is produced through microbial use of oxygen bound in sulphate. The hydrogen sulphide concentration is counted as negative oxygen. In the case of oxic near-bottom conditions, a fixed portion of nitrogen recycled in the sediments is removed from the system through consecutive nitrification and denitrification. The model accounts for the oxygen-dependent dynamics of phosphate in sediments: under oxygenated conditions, part of the mineralised phosphate is forming iron-phosphate complexes which are stored in the sediments, whereas in anoxic conditions the previously stored phosphate is liberated to the overlying water. Detailed description and formulation of the model is given in [28]–[30].

**Figure 1:**
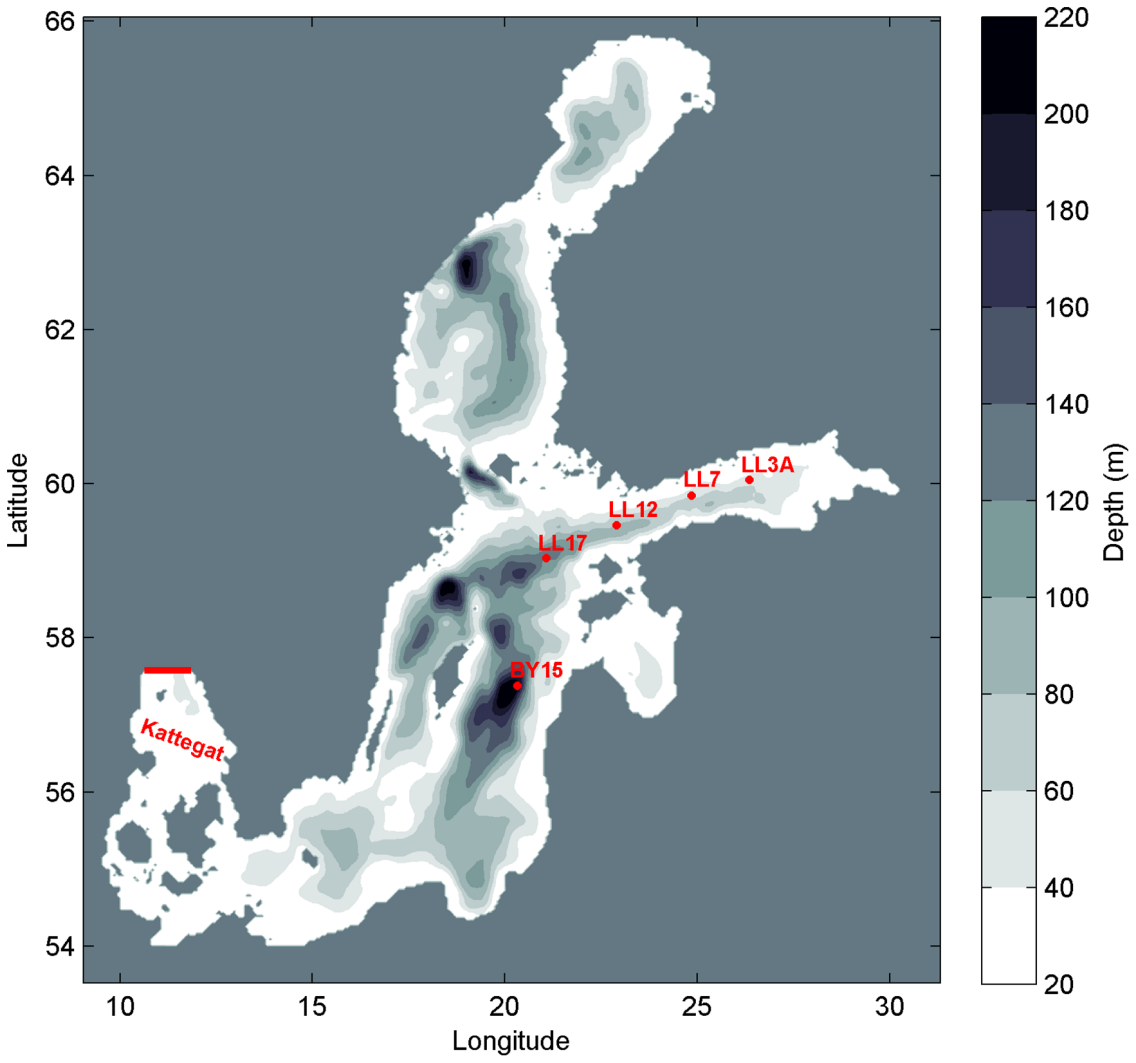
Bathymetric map of the model domain. Locations of monitoring stations BY15 (57.32°N, 20.05°E, depth 238 m), LL17 (59.03°N, 21.08°E, depth 171 m), LL12 (59.48°N, 22.9°E, depth 82 m), LL7 (59.85°N, 24.84°E, depth 100 m) and LL3A (60.07°N, 26.35°E, depth 68 m) are shown. Location of the model open boundary in Kattegat is indicated by red line.

The model domain covers the entire Baltic Sea area with an open boundary in the northern Kattegat (Fig. 1). Bathymetry was interpolated to a 2×2 nm (3704×3704 m) model grid from the digital topography of the Baltic Sea [31]. 25 layers were applied in the vertical, using adaptive coordinates. Adaptive coordinates are based on a vertical optimization of the layer distribution which depends on vertical density and velocity gradients and the distance to surface and bottom [20]. The time step implemented is 30 s for the barotropic and 600 s for the baroclinic mode. The period modelled is 01.01.1990–31.12.2009. During the first year of the simulation only hydrodynamics was modelled as a spin-up for the coupled hydrodynamic-biogeochemical simulation.

Initial distributions of water temperature and salinity for January 1990 were interpolated to the model grid from the monthly climatological data set [32]. Initial distributions of nitrate, ammonium, phosphate and dissolved oxygen were reconstructed from a limited amount of available measurement data covering the winter of 1991 [33] and interpolated to the model grid. All the other biogeochemical model variables were given uniform initial distributions over the model domain based on previously reported typical winter values. Prescribed salinity and temperature distributions at the open boundary were interpolated using monthly climatological data [32]. Hourly sea level fluctuations at the open boundary were interpolated from gauge measurements at Kattegat.

The model was forced with European Centre for Medium Range Weather Forecasting (ECMWF) ERA-Interim reanalysis meteorological data. The ERA-Interim configuration uses a 30 min time step and has a spectral T255 horizontal resolution, which corresponds to approximately 79 km spacing on a reduced Gaussian grid [34]. The original data on air temperature, dew point temperature, air pressure, cloud cover, wind speed and wind direction, were interpolated to a regular Gaussian grid corresponding to approximately 50 km spacing with 6-hourly temporal resolution. The model took into account land-based runoff and nutrient loads which had been incorporated into 20 major rivers [30]. Atmospheric deposition of nutrients was taken constant over the entire modelled period.

The setup of the experimental run was identical to the reference run as described above. The only difference being that sea level fluctuations at the open boundary were not prescribed, thus suppressing one of the major factors driving the barotropic inflows to the Baltic Sea [35].

## Results

### Validation of the reference run

The performance of the model was validated at locations corresponding to HELCOM monitoring stations BY15 (57.32°N, 20.05°E, depth 238 m), LL17 (59.03°N, 21.08°E, depth 171 m), LL12 (59.48°N, 22.9°E, depth 82 m), LL7 (59.85°N, 24.84°E, depth 100 m) and LL3A (60.07°N, 26.35°E, depth 68 m), representing a transition path from the Baltic Proper (Gotland Deep) to the central-eastern parts of the Gulf of Finland.

To summarize the model performance over the whole period, the modelled and measured salinity, temperature, nitrate, ammonium, phosphate and dissolved oxygen at the sea surface and at depths of 20 m, 50 m and (if applicable) 100 m as well as near the bottom were compared at all stations using a cost function, formulated as CF  =  |(M–D)/SD|, where the bias (M–D) of the model mean (M) relative to the mean of observations (D) is normalized to the standard deviation (SD) of the observations. Cost function values 0–1 indicate a good match between the model results and measurements, 1–2 indicate a reasonable match and values above 2 indicate a poor match [36]. Among the parameters compared, modelled temperature follows measured values most accurately, followed by oxygen and inorganic nutrients, while salinity has slightly higher cost function values in the upper layers (Table 1).

**Table 1:** Cost function values for salinity (PSU), temperature (°C), nitrate (mmol N/m^3^), phosphate (mmol P/m^3^), oxygen (ml/l) and ammonium (mmol N/m^3^) at the surface (S), 20 m, 50 m and 100 m depths and near the bottom (B) at stations LL3A, LL7, LL12, LL17 and BY15.

		S	20	50	100	B
Salinity	**LL3A**	1.82	0.61	0.19		0.52
	**LL7**	0.93	1.63	0.26		0.62
	**LL12**	1.35	2.46	1.02		0.32
	**LL17**	1.27	1.42	0.27	0.65	0.93
	**BY15**	1.42	1.20	0.98	0.70	0.32
Temperature	**LL3A**	0.24	0.26	0.07		0.10
	**LL7**	0.19	0.01	0.12		0.14
	**LL12**	0.19	0.00	0.11		0.33
	**LL17**	0.17	0.31	0.09	0.65	0.90
	**BY15**	0.16	0.47	0.22	0.75	0.79
Nitrate	**LL3A**	0.34	0.01	1.89		1.36
	**LL7**	0.29	0.09	0.60		0.67
	**LL12**	0.06	0.14	0.07		0.74
	**LL17**	0.07	0.22	0.79	0.06	0.23
	**BY15**	0.03	0.35	1.21	0.61	1.24
Phosphate	**LL3A**	1.26	1.42	1.47		0.68
	**LL7**	0.89	0.86	1.70		0.08
	**LL12**	0.81	0.56	0.45		0.50
	**LL17**	0.02	0.03	0.09	0.30	0.09
	**BY15**	0.32	0.23	0.33	0.63	0.90
Oxygen	**LL3A**	0.29	0.81	0.84		0.86
	**LL7**	0.04	0.42	0.81		0.21
	**LL12**	0.10	0.58	0.67		0.75
	**LL17**	0.11	0.85	0.84	0.34	0.50
	**BY15**	0.17	1.08	1.55	0.06	0.89
Ammonium	**LL3A**	0.20	0.57	1.70		0.99
	**LL7**	0.33	0.15	0.35		0.53
	**LL12**	0.55	0.11	0.56		0.68
	**LL17**	0.50	0.09	0.54	0.84	1.13
	**BY15**	0.63	0.50	0.47	0.30	1.08

Cost function values 0–1 indicate a good result, 1–2 indicate a reasonable result and >2 indicate a poor result.

Agreement of the temporal dynamics of modelled salinity, temperature, oxygen, nitrate and phosphate with corresponding measurement data for the near-bottom layer (ranging 0–2 m from the sea floor) of the northern Baltic Proper (station LL17) and the western Gulf of Finland (station LL12) was investigated.

**Figure 2:**
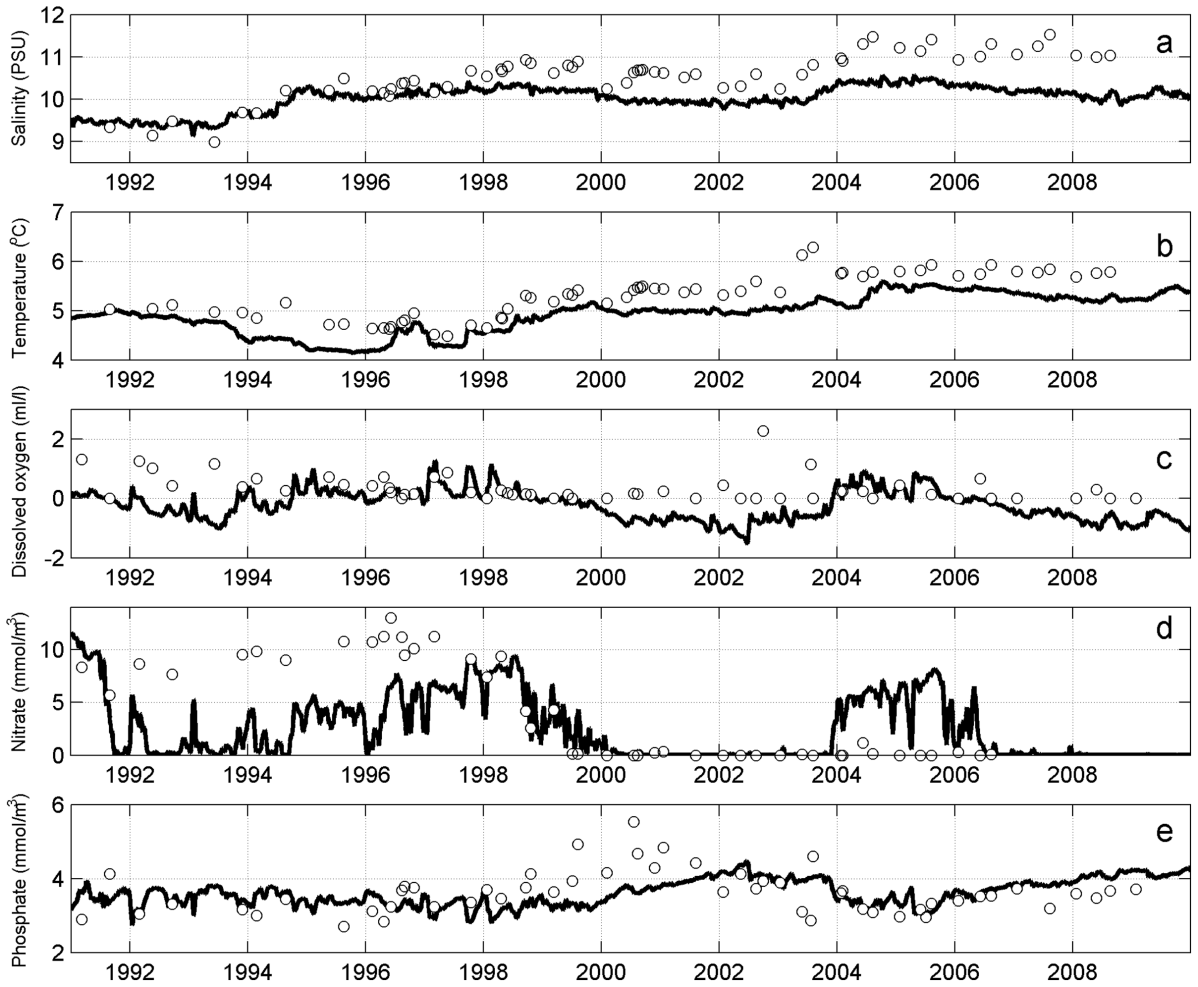
Comparison of modelled (lines) and monitored (circles) near-bottom salinity (a), temperature (b), dissolved oxygen (c), nitrate (d) and phosphate (e) at station LL17.

At station LL17 (Fig. 2) near-bottom salinity was modelled most accurately during the years 1991–1996. An increase of around 1 PSU caused by the MBIs of 1993–1994 was properly reproduced. However, the effect of the subsequent inflow events was underestimated by the model, which by the end of 2008 led to a modelled salinity of about 1 PSU lower than in the measurements. In general, the model underestimated deep water temperature by a margin of up to 0.5°C, with the exception of the year 2003 when the difference between the measured and modelled temperatures was 1°C. Temporal course of the near-bottom temperature was simulated rather well, with a decreasing trend lasting up until 1997, an increasing one from 1997 to 2005 and a stable temperature in the final period of the model. Both model and measurement oxygen data confirmed that at this station either anoxic or hypoxic (oxygen concentrations 0–2 ml/l, see [22]) conditions were dominant near the bottom. While model results showed anoxic conditions during 1991–1995, measurements indicate a presence of oxygen for this time period, which might be explained by uncertainty in the initial distributions of the biogeochemical variables in the model. It must be noted that negative oxygen concentrations shown in the model results mean that there is a presence of H_2_S and in the current context represent the severity of anoxia. In the measurement data the absence of oxygen is indicated by zero oxygen concentrations. The temporal evolution of near-bottom nitrate was in general well-reproduced: it is present most of the time up to the year 2000 and absent thereafter. Near-bottom nitrate dynamics are closely dependent on oxygen concentrations, and therefore the mismatch between modelled and measured nitrate (i.e. too low nitrate during 1991–1997 and too high around 2005) is caused by mismatch between modelled and measured oxygen concentrations. The model accurately reproduced phosphate dynamics, where after the onset of anoxic conditions it showed an increase in phosphorus concentrations caused by the release of phosphate which was previously stored in the sediments.

**Figure 3:**
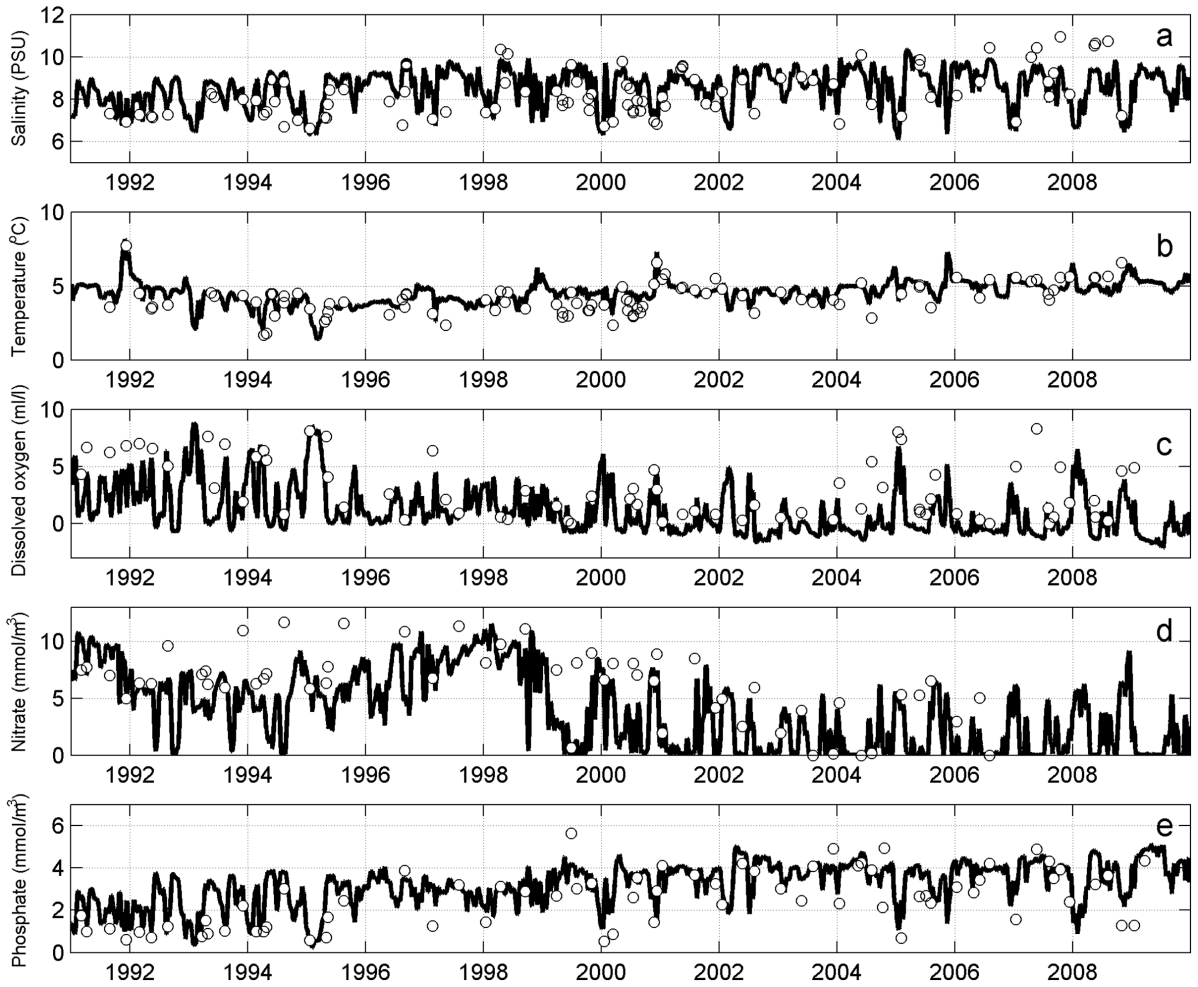
Comparison of modelled (lines) and monitored (circles) near-bottom salinity (a), temperature (b), dissolved oxygen (c), nitrate (d) and phosphate (e) at station LL12.

At station LL12 in the western part of the Gulf of Finland (Fig. 3) the model accurately reproduced the steady increase of near-bottom salinity and its variability during the course of the investigated period. Near-bottom temperature was rather accurately simulated by the model, capturing intra-annual variability. Simulated oxygen followed the measurement data rather closely, reproducing the shift from well-oxygenated (concentrations of up to about 8 ml/l) to mostly hypoxic and anoxic near-bottom conditions that started from 1995 as well as the increase in variability since 2004. Near-bottom nitrate concentrations were high during the first half of the modelled period, while in the second half concentrations of nitrate showed high fluctuations and nitrate-depleted conditions were present frequently. Compared to the measurement data, the model somewhat underestimated nitrate concentrations in the mid-1990s. Near-bottom nitrate variability clearly reflects its dependence on oxygen dynamics. The modelled phosphate matched the measurement data rather accurately, as an increase over the modelled period and small-scale variability in concentrations were correctly reproduced.

Validation and detailed analysis of the results of the model for the central Gulf of Finland (HELCOM station LL7) are given in [37].

### Results of the experimental run

**Figure 4:**
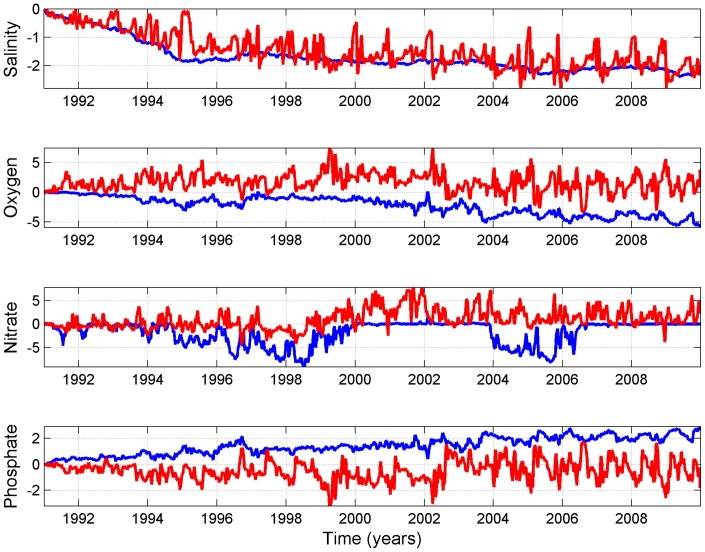
Difference between results of reference and experimental run for near-bottom salinity (PSU), oxygen (ml/l), nitrate (mmol N/m^3^) and phosphate (mmol P/m^3^) at station LL17 (blue lines) and LL12 (red lines).

The effect of eliminating highly saline water inflows on the near-bottom conditions at stations LL17 and LL12 was analysed by subtracting the daily mean values of the reference run from the results of the experimental run (Fig. 4). Over the entire modelled period the salinity was lower in the experimental run than in the reference run. Temporal dynamics was similar at both stations, but at LL17 the mean difference between the two runs was slightly higher and showed less small-scale variability than at LL12. In the absence of saltwater inflows, near-bottom oxygen at LL17 decreased until the difference between the two runs reached about 5 ml/l at the end of the modelled period. At LL12 the experimental run showed a general improvement of oxygen conditions, but the variability was high and there were short periods when the oxygen concentrations were lower than in the reference run. This occurred more often during the last third of the modelled period. At station LL17 near-bottom nitrate in the experimental run was close to or lower than in the reference run, reflecting both faster consumption of nitrate in the first half of the modelled period and the fact that as a result of stronger anoxia nitrate was later completely depleted. At station LL12 the opposite situation occurred: in the experimental run nitrate was usually more abundant, especially during the second half of the modelled period, although the difference was variable due to frequent changes in oxygen conditions. The difference in near-bottom phosphate at station LL17 increased steadily over the modelled period, reaching about 2.5 mmol/m3 higher concentration in the experimental run. At LL12 the experimental run showed a decrease in mean phosphate until the year 2002, thereafter variability of phosphate increased (the difference varying approximately from −2 to 2 mmol/m3) and, while the mean was still lower in the experimental run, higher phosphate concentrations than in the reference run were often present.

### Near-bottom N:P ratio

**Figure 5:**
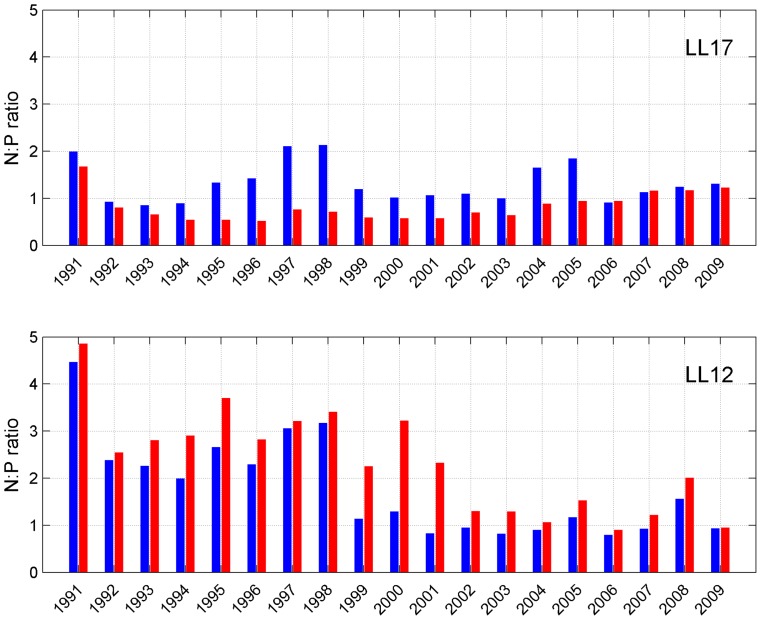
Yearly mean N:P ratios at stations LL17 and LL12 in the reference run (blue bars) and experimental run (red bars). N represents the sum of nitrate and ammonium concentrations.

To summarize the effect of the absence of MBIs and the resulting lower water salinity on near-bottom nutrient content, yearly mean inorganic nitrogen to phosphate (N:P) ratios (N representing the sum of nitrate and ammonium) for near-bottom layers in both the reference and experimental runs were calculated (Fig. 5). At station LL17 N:P ratio in the reference run was up to 2–3 times higher than in the experimental run, except for the last four years, when N:P ratios in both runs were very close to each other. In 2006 and 2007 N:P ratio was even slightly higher in the experimental run. N:P ratio decreases during the first half of the modelled period in the experimental run and subsequently increases due to higher near-bottom ammonium concentrations under anoxic conditions.

At station LL12 N:P ratio was always higher for the experimental run. The ratio was relatively high up to the year 1998 in the reference run and up to 2000 in the experimental run and in both cases decreased thereafter due to the onset of anoxic conditions near the bottom. Due to the sporadic character of short-lasting periods of anoxia, N:P ratio in the experimental run remained slightly higher until the end of the modelled period.

### Comparison of variability of near-bottom oxygen and salinity

The dynamics of the variability of near-bottom oxygen concentrations in relation to salinity variations at stations LL17 and LL12 were analysed for the reference and the experimental runs. Table 2 summarizes the correlation coefficients of daily mean salinity and oxygen for each modelled year and for the total simulation period calculated at 95% confidence level.

**Table 2:** Correlation coefficients of mean near-bottom salinity and oxygen for each modelled year and the total simulation period at stations LL17 and LL12 in the reference run (ref) and experimental run (exp).

Year	LL17 ref	LL17 exp	LL12 ref	LL12 exp
**1991**	0.27	0.69	−0.92	−0.89
**1992**	0.10	0.64	−0.94	−0.95
**1993**	0.50	−0.03	−0.97	−0.97
**1994**	0.53	−0.21	−0.91	−0.87
**1995**	0.42	−0.78	−0.97	−0.94
**1996**	0.43	0.17	−0.90	−0.84
**1997**	0.48	0.29	−0.79	−0.78
**1998**	0.16	0.03	−0.89	−0.88
**1999**	0.61	−0.25	−0.94	−0.89
**2000**	0.73	0.22	−0.95	−0.91
**2001**	0.42	−0.43	−0.87	−0.84
**2002**	0.66	−0.54	−0.96	−0.98
**2003**	0.63	−0.86	−0.82	−0.76
**2004**	0.47	−0.47	−0.82	−0.87
**2005**	0.72	−0.22	−0.90	−0.96
**2006**	0.74	−0.50	−0.83	−0.95
**2007**	0.33	−0.29	−0.88	−0.93
**2008**	0.79	−0.05	−0.88	−0.94
**2009**	0.74	−0.24	−0.83	−0.95
**total**	0.40	0.74	−0.84	−0.60

**Figure 6:**
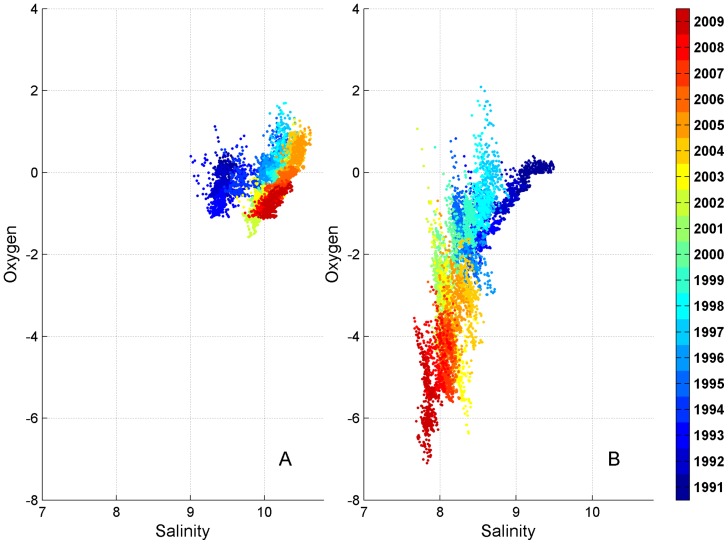
Scatter plots of near-bottom salinity vs oxygen at station LL17 in the reference (A) and experimental (B) runs. Individual years are represented by distinct colours.

At station LL17 salinity and oxygen variability in the reference run was lower than in the experimental run (Fig. 6a). Before the occurrence of the first MBI in 1993, salinity was mostly in the range between 9 to 10 PSU, while oxygen concentration was between −1 and 1 ml/l. As a consequence of the MBIs, the following years were characterized by a shift towards slightly higher salinity, up to approximately 10.5 PSU, and slightly increased oxygen concentrations. On the other hand, during the following more stagnant years a slight salinity decrease (down to 9.5 PSU) and lowering of oxygen content were evident. Nevertheless, oxygen always stayed in the range of −2 to 2 ml/l. High positive correlation coefficients were characteristic for the periods of MBIs (e.g. 1993–1994) and stagnant years (e.g. 1999–2000). In the first case they were caused by the inflow of saline and relatively oxygenated water and in the second case by steady decrease of salinity and loss of oxygen. Years with relatively stable conditions (e.g. 1998, 2007) had a lower correlation coefficient of salinity and oxygen concentrations. If we consider the entire modelled period as one data set, then the correlation coefficient between salinity and oxygen, similarly to the correlation coefficients of individual years, is positive.

In the experimental run at station LL17 there was a more significant drop in salinity (from around 9.5 to 7.6 PSU) and a shift towards more severe oxygen depletion (from slightly above 0 to around −7 ml/l) over the course of the modelled period (Fig. 6b). The correlation coefficient between salinity and oxygen was above 0.5 only during the first two simulation years, due to the decrease of both salinity and oxygen. While there were some years with low but positive correlation coefficient values, most of the years were characterized by a negative correlation coefficient explained by the supply of relatively more saline but oxygen-depleted water from the deeper areas of the Baltic Proper and its subsequent mixing with less saline and relatively oxygenated (or less oxygen-depleted) water from the overlying layers. Since in this run salinity in the Baltic Proper is not compensated by inflows through the Danish Straits, the system experiences yearly shifts towards lower salinity and stronger anoxia. Therefore, strong positive correlation (0.74) exists for the entire modelled period, although correlation coefficients for most of the individual years were negative.

**Figure 7:**
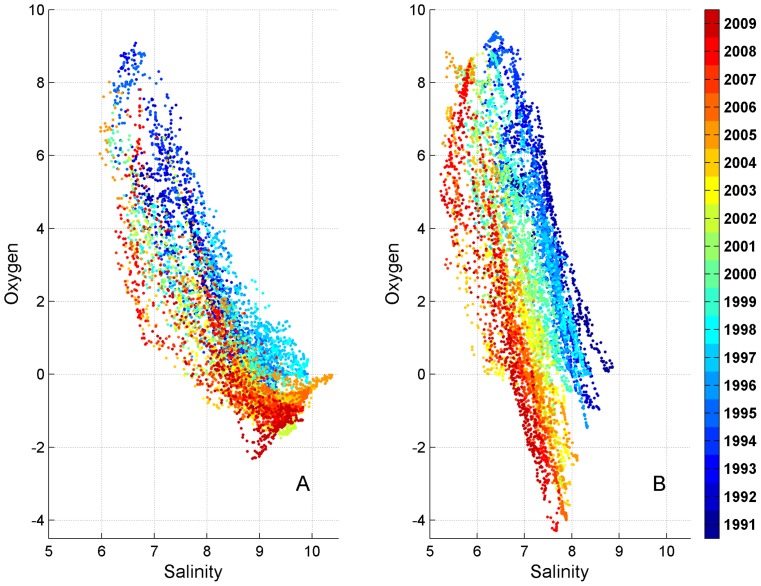
Scatter plots of near-bottom salinity vs oxygen at station LL12 in the reference (A) and experimental (B) runs. Individual years are represented by distinct colours.

In the reference run, station LL12 was characterized by variations of salinity from around 6 to 10.5 PSU, and of oxygen from around −2.5 to 9 ml/l (Fig. 7a). Compared to the Northern Baltic Proper area, there was a high intra-annual variability in both parameters. Every individual year was characterized by a strong negative correlation between salinity and oxygen. High salinity is associated with low oxygen or anoxia, while lower salinity is connected to the occurrence of higher oxygen concentrations at the near-bottom. However, at salinity higher than 10 PSU no decrease in oxygen was seen, indicating that in case of favourable conditions intense water inflows are able to import oxygenated water to the western Gulf of Finland from deeper areas of the Baltic Sea. The correlation coefficients between salinity and oxygen during individual years were close to the correlation coefficient for the entire period (−0.84).

In the experimental run, salinity ranged from about 5 to 9 PSU and oxygen from −4 to almost 10 ml/l at station LL12 (Fig. 7b). Similarly to the reference run, individual years were characterized by negative correlations of salinity and oxygen. However, in comparison to the reference run, decreased saltwater supply from the Baltic Proper leads to a decreased stratification of the water column. As a consequence, the salinity becomes gradually lower over the course of the entire modelled period, while oxygen values remain relatively stable. This results in weaker correlation for the entire modelled period (−0.6), than for each individual year (∼−1).

## Discussion

Validation of the reference run showed that the model rather accurately reproduced hydrodynamic and biogeochemical conditions in the study area. Eilola et al [38] evaluated biogeochemical cycles in three state-of-the art numerical models of the Baltic Sea and presented long-term (1970–2005) cost-function values at 6 stations, which included 2 of the stations evaluated in the present paper, namely BY15 (Gotland Deep) and LL7 (central Gulf of Finland). Although the modelled period was shorter in the present study, the quality of the results reproduced is comparable to those presented in [38].

The mismatch between the modelled parameters and observed values can be explained by uncertainties in the setup, initial conditions and forcing of the hydrodynamic model as well as from the uncertainties in parameterization of biogeochemical processes. Meier et al [39] showed that a horizontal resolution of 2 nm was necessary for the model to reproduce the January 1993 saltwater inflow to the Baltic Sea. Near-bottom salinity in the deep areas of the Baltic Sea after the first inflow events was underestimated in our model. This issue could be solved by further refining the setup of the hydrodynamic model. A robust example of the influence of uncertainties in hydrodynamic modelling on biogeochemical cycling is the presence of substantial concentrations of nitrate in the near-bottom layers at station LL17 in the model results for 2004–2006. This was caused by propagation of less dense but oxygenated water along the bottom, which triggered the nitrification of ammonium. Comparison of time-series of near-bottom variables at LL17 and LL12 leads to the conclusion that uncertainties in setup and forcing have higher effect at deeper stations due to longer water residence time and weaker response to the variability of external forcing.

Accurate representation of hydrodynamical processes is a prerequisite for credible simulations of biogeochemical cycling in a coupled 3D ecosystem-physical model. This is especially valid for the Baltic Sea, where ecosystem dynamics are strongly controlled by physical processes. Due to the differences in depth and vertical density stratification between central Baltic Sea and its sub-basins, the biogeochemical response of these regions to decreasing salinity can differ significantly.

Results of the experimental run showed decreasing near-bottom salinity at both stations. However, the response of biogeochemical variables was different in the north-eastern (NE) Baltic Proper and in the western Gulf of Finland. In the absence of strong inflows the near-bottom layer at station LL17 became increasingly anoxic as time passed, while at the shallower station LL12 mean near-bottom oxygen level increased due to weaker stratification and consequent stronger mixing throughout the water column. On the other hand, starting from the year 2002, more severe anoxia in the Baltic Proper was also manifested at LL12 in the form of frequent but short-lasting events of oxygen depletion. Inflows of saline and oxygen-depleted water from NE Baltic Proper to the Gulf of Finland usually take place due to the typical estuarine circulation in the Gulf. Liblik et al [40] observed that the deep water salt wedge in winter 2012 originated from the Baltic Proper at a depth range of 110–115 m and concluded that deterioration of deep layer oxygen conditions was solely related to the advective transport of hypoxic water from the NE Baltic Proper. In the case of a weaker and deeper halocline in the Baltic Proper, oxygen conditions in the Gulf of Finland improve. As confirmed by the results of experimental run, the mean near-bottom oxygen concentration in the western Gulf increases in the absence of MBIs, but after several years of stagnant conditions anoxia in the deeper layers of the Baltic Proper becomes more severe and estuarine transport leads to the emergence of short-lasting events of deterioration of water quality (lower oxygen or anoxia and higher phosphate concentrations) at the near-bottom of the western Gulf of Finland. Extrapolating our results beyond the modelled period, further deepening of the halocline in the Baltic Proper is likely to prevent the inflows of anoxic water to the Gulf of Finland and will lead to improved near-bottom conditions in the long term.

This is in accordance with the study of Gustafsson et al [41] who modelled the influence of several engineering measures which aimed to reduce the effects of eutrophication of the Baltic Sea. Some of the measures were connected to the management of the flow capacity of the Danish Straits, e.g. closing Öresund at the Drogden Sill. This scenario showed the presence of a long (lasting for more than 30 years) transitional period of stagnation in the Baltic Proper, during which hypoxia increased in deeper waters. After this period the water quality improved and salinity was extremely reduced [42]. Our modelling experiment confirms that within the time frame of simulation deep areas of the Baltic Proper would become more anoxic in the absence of strong saltwater inflows, while in the shallower areas (western Gulf of Finland) near-bottom conditions would improve on average. Thus, in most cases the improvement of near-bottom oxygen conditions following the decrease in saltwater inflows through the Danish Straits initially takes place in the shallower areas of the Baltic and then propagates to the deeper areas as water becomes fresher and stratification becomes weaker.

Experimental run results showed a rapid response in near-bottom dynamics of nitrate and phosphate to the variability in oxygen. At LL17 nitrate was consumed faster than in the reference run, while at LL12 mean nitrate concentrations increased, yet variability was high with sporadic periods of depletion in response to the rapidly changing oxygen conditions. The lack of difference between nitrate in the experimental and reference runs during 2000–2004 can be explained by the absence of nitrate in both cases, and the difference in 2004–2006 is an artefact of overestimated nitrate in the reference run during the same period. Compared to the reference run, near-bottom phosphate at LL17 increased over the course of the modelling period due to the lack of phosphate binding to the sediments under anoxic conditions. At station LL12 better oxygen conditions led to decreasing phosphate concentrations in the water column. After 2002, mean phosphate concentrations in the experimental run slightly increased due to the enhanced frequency of short-lasting events of anoxic water inflow from the Baltic Proper which caused the release of phosphate from sediments into the overlying water. Therefore, in the time frame of the simulated period, the absence of strong saltwater inflows and the consequent freshening of the Baltic Sea led to opposite responses in near-bottom inorganic nutrients in the Baltic Proper and the Gulf of Finland. However, despite the decrease and depletion of nitrate and increase of phosphate, near-bottom N:P ratios in the experimental and the reference runs were rather similar and even increased slightly towards the end of the simulation period. Although there was more phosphate at LL17 in the experimental run, nitrogen concentration (in the form of ammonium) was also higher than in the reference run. In the western Gulf of Finland N:P ratio started to decrease from the year 1999 in the reference run and from 2001 in the experimental run. This is clearly correlated to the change in deep oxygen conditions. However, since 2002 N:P ratio in the experimental run only slightly exceeded the reference run values. This can be attributed to the emergence of inflows of anoxic water from the Baltic Proper starting from that year. Phosphate release from sediments occurs rapidly after sediments become anoxic, but phosphate binding takes place at a slower pace following the deposition and the subsequent mineralization of organic matter. Kahru et al [8] reported the decrease of N:P ratio starting from 1995 in the Gulf of Finland, which was possibly triggered by the major saline water inflow in 1993. Results of our experiment show that the decrease in near-bottom N:P ratio takes place also in the absence of Major Baltic Inflows. However, in that case it is caused by inflowing anoxic water from NE Baltic Proper to the Gulf of Finland, releasing phosphate previously bound to the sediments and the simultaneous decreasing nitrate due to denitrification.

Nutrient dynamics in the deep layers of the Baltic Sea depends to a large extent on the dynamics of salt and oxygen. Analysis of correlation of the two provides a better understanding of the origin of water masses and their impact on the governing biogeochemical conditions. Salinity-oxygen scatter plots for station LL17 show that near-bottom oxygen conditions in that area improve as a consequence of oxygen-rich saltwater inflows. In the case of stagnant conditions together with decreasing salinity also more severe anoxia is expected. Although stratification becomes weaker, mixing processes are still not intensive enough to improve near-bottom oxygen conditions. The situation is different in the shallower western Gulf of Finland area. Vertical mixing as well as estuarine transport reversals are able to increase near-bottom oxygen concentrations to 8–9 ml/l, which is typical in surface water [40],[43]. This process is more easily achieved in the conditions of the less stratified water column of the experimental run. In the reference run, during the usual estuarine transport in the Gulf of Finland, near-bottom anoxia becomes slightly less pronounced after the inflows of highly saline deep water (>9 PSU), which bring oxygen originating from the water masses of the Major Baltic Inflows. In the experimental run this time period is characterized by the most severe anoxic conditions despite lower salinity and density stratification.

The dynamic nature of external forcing, leading to a spatially variable biogeochemical response, makes the Baltic Sea unique amongst regions with the occurrence of deep water hypoxia and/or anoxia. For instance, in case of the northern Gulf of Mexico it was found that chemical-biological processes were mainly responsible for maintaining anoxic near-bottom conditions [44]. Similarly, water column models simulating biogeochemical cycles in regions where hypoxic conditions are permanently present (for example, the highly stratified Black Sea), indicate the importance of organic matter supply and decomposition on the structure of the redox interface and the corresponding processes [45]. In contrast, the occurrence of large oxygen minimum zones is considered to be primarily caused by weak ocean ventilation through subsurface currents [46].

Since ocean warming and increased stratification caused by climate change will likely reduce deep-water oxygen concentrations in the future, Peña et al [47] stressed urgency to develop models coupling realistic physics and biogeochemistry at appropriate scales. For the Baltic Sea region it is expected that climate change will decrease frequency of MBIs and increase run-off due to higher precipitation. Decreasing salinity stratification will have important implications for the ecosystem of the entire Baltic Sea, as shown in our modelling study.

We propose the approach of comparing results of a hindcast scenario (reference run) and its modified versions with controlled alterations in e.g. external forcing, initial conditions or process rates (experimental run). Such an approach can be credibly used to further improve our understanding of marine system functioning, and its response patterns to global and regional changes and management measures. In contrast to future scenario modelling, this method is more substantiated because the results of hindcast simulations are validated against observational data so that robust quantitative comparisons between default and perturbed cases can be made.

## Conclusions

A coupled three-dimensional hydrodynamic-biogeochemical model was set up and applied for the Baltic Sea area with an aim to simulate the changes in salinity, nutrients and oxygen dynamics during 1991–2009, focussing on the northern Baltic Proper and the western Gulf of Finland. Using available measurement data, validation showed that the model performance was rather accurate. In order to investigate the effects of MBIs on hydrophysical and biogeochemical conditions in the focus area, we compared the results of the reference run with the results of the experimental run where inflows of highly saline water to the Baltic Sea from the North Sea were suppressed. Results of the experimental run showed decreasing near-bottom salinity at both stations LL17 and LL12. In the course of the modelled period anoxia became stronger at deep station LL17, while shallower station LL12 showed an average increase in near-bottom oxygen levels due to weaker stratification and intensified water mixing. On the other hand, compared to the reference run at station LL12, emergence of short-lasting events of lower oxygen were associated with the estuarine transport of anoxic water masses from the Baltic Proper. Consequently, during the second half of the modelled period near-bottom N:P ratios decreased in the absence of MBIs both in the stagnant Baltic Proper and in the western Gulf of Finland. Our results confirmed that accurate representation of MBIs in hydrodynamic models is important for realistic simulations of biogeochemical properties of both deep central areas and shallower sub-basins of the Baltic Sea.
